# Replacing a Palatable High-Fat Diet with a Low-Fat Alternative Heightens κ-Opioid Receptor Control over Nucleus Accumbens Dopamine

**DOI:** 10.3390/nu13072341

**Published:** 2021-07-09

**Authors:** Conner W. Wallace, Nari S. Beatty, Sarah A. Hutcherson, Heather A. Emmons, Madison C. Loudermilt, Steve C. Fordahl

**Affiliations:** The Department of Nutrition, The University of North Carolina at Greensboro, 319 College Ave, 318 Stone Building, Greensboro, NC 27412, USA; cwgleaso@uncg.edu (C.W.W.); nsbeatty@uncg.edu (N.S.B.); sahutche@uncg.edu (S.A.H.); haemmons@uncg.edu (H.A.E.); mclouder@uncg.edu (M.C.L.)

**Keywords:** κ-opioid receptors, high-fat diet replacement, dopamine neurotransmission, nucleus accumbens core, novelty-induced hypophagia, elevated plus-maze, fast-scan cyclic voltammetry, anxiety-like behavior, diet-induced obesity

## Abstract

Diet-induced obesity reduces dopaminergic neurotransmission in the nucleus accumbens (NAc), and stressful weight loss interventions could promote cravings for palatable foods high in fat and sugar that stimulate dopamine. Activation of κ-opioid receptors (KORs) reduces synaptic dopamine, but contribution of KORs to lower dopamine tone after dietary changes is unknown. Therefore, the purpose of this study was to determine the function of KORs in C57BL/6 mice that consumed a 60% high-fat diet (HFD) for six weeks followed by replacement of HFD with a control 10% fat diet for one day or one week. HFD replacement induced voluntary caloric restriction and weight loss. However, fast-scan cyclic voltammetry revealed no differences in baseline dopamine parameters, whereas sex effects were revealed during KOR stimulation. NAc core dopamine release was reduced by KOR agonism after one day of HFD replacement in females but after one week of HFD replacement in males. Further, elevated plus-maze testing revealed no diet effects during HFD replacement on overt anxiety. These results suggest that KORs reduce NAc dopamine tone and increase food-related anxiety during dietary weight loss interventions that could subsequently promote palatable food cravings and inhibit weight loss.

## 1. Introduction

Obesity prevalence has steadily increased for decades with 42.4% of American adults classified as obese [1]. Westernized food environments contribute to obesity by promoting palatable foods high in energy density from sugar and fat. To compound this, a recent systematic review found less than half of research participants could maintain weight loss, and psychological tension generated by lifestyle changes promoted relapse to obesogenic habits [2]. Further, animal models showed that replacement of a diet high in saturated fat (HFD) with low-fat food induced anxiety-like behavior and motivation to obtain sucrose and fat [3,4]. This suggests that extended palatable food consumption promotes dietary inflexibility towards non-preferred foods but sudden removal of HFD induces stress and motivation to consume palatable options.

Food is a natural reward that activates the dopamine system through its caloric and sensory properties [5,6]. Sudden dietary changes that promote stress and food intake could be explained by dopamine neurotransmission in the nucleus accumbens (NAc) that controls cue-induced learning and motivation [7]. Dopamine concentrations in the medial NAc core and shell increase acutely in response to energy value and orosensory properties of sucrose [5] and fat [6], and phasic dopamine release in the core was promoted by cues predictive of sucrose versus non-caloric saccharin [8]. Conversely, chronic intake of palatable foods may reduce dopamine neurotransmission, as extended intake of a HFD reduced capacity for phasic dopamine release [9], delayed dopamine reuptake [9,10,11], and altered expression of dopamine receptors (D1R and D2R) and the dopamine transporter (DAT) [12,13]. NAc dopamine release and dopamine neuron firing are reduced by presynaptic κ-opioid receptor (KOR) signaling [14,15,16], and synaptic dopamine is further controlled by DAT shuttling and DAT-mediated dopamine reuptake induced by D2Rs and KORs [17,18,19]. Diet-induced obesity reduces tonic levels of dopamine in the dorsal striatum and inhibits striatal activation during consumption of palatable foods [20,21] but heightens activation upon exposure to palatable food cues [21]. KORs reduce synaptic dopamine concentrations [16,17,19], but the function of KORs in altering NAc dopamine neurotransmission after stressful dietary changes is unknown. We propose that replacement of a HFD with a low-fat control diet would heighten KOR sensitivity to further reduce dopamine tone.

Mechanisms that contribute to anxiety and disrupt dopamine neurochemistry after dietary changes and weight loss are unclear. Therefore, the purpose of this study was to examine food intake behavior and diet-induced anxiety, and to characterize KOR-mediated control over dopamine neurotransmission in the NAc core after HFD was replaced with a low-fat diet. Specifically, we examined responsivity to KOR agonism and antagonism and compared differences due to length of HFD replacement, diet, and sex. We hypothesized that HFD replacement would reduce dopamine release and reuptake measured with fast-scan cyclic voltammetry (FSCV) and potentiate effects of KOR agonism to reduce dopamine release but attenuate effects of KOR antagonists. We further predicted that HFD intake would reduce preference for sucrose during novelty-induced hypophagia (NIH) and increase anxiety-like behavior during the elevated plus-maze (EPM). Our data suggest that males responded more strongly to KOR stimulation after one week of HFD replacement, whereas females responded acutely after one day of HFD replacement. Importantly, we highlight effects of diet, sex, and HFD replacement on NAc KOR sensitivity and behavior.

## 2. Materials and Methods

### 2.1. Animals, Diet, and Experimental Design

**Figure 1:**
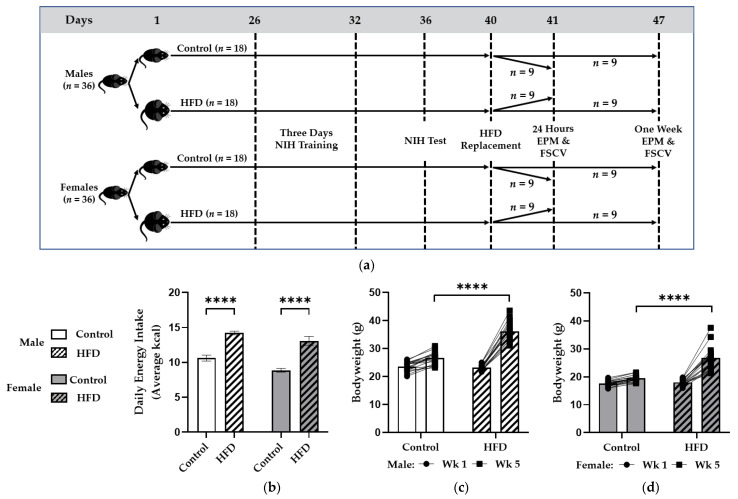
Experimental Design, Food Intake, and Bodyweight. (**a**) Male and female C57BL/6J mice were placed on a control 10% fat diet (Control) or high saturated 60% fat diet (HFD) for six weeks. During ad libitum feeding, the novelty-induced hypophagia (NIH) paradigm was completed. Subsequently, the HFD replacement paradigm was started and continued for one day or one week, followed by the elevated plus-maze (EPM) then ex vivo slice fast-scan cyclic voltammetry (FSCV) immediately afterwards. Average daily kcal intake (**b**) is shown for all groups in addition to bodyweight in grams at weeks one (Wk 1) and five (Wk 5) of feeding for males (**c**) and females (**d**). Bodyweight data are displayed individually for each mouse. * denotes a significant difference between diet groups. ****, *p* < 0.0001.

Methods were approved by the Institutional Animal Care and Use Committee of the University of North Carolina at Greensboro (protocol code 19-004). Six-week-old C57BL/6 male (*n* = 36) and female (*n* = 36) mice purchased from Jackson Laboratories (Bar Harbor, ME) were housed 3/cage on a 12 h light/dark cycle (lights off 0600, lights on 1800). Mice had free access to water and either control diet (DIO series D12450K, Research Diets Inc.) containing 10% total kcals from fat with 3.85 kcals/g or a high-saturated-fat diet (HFD) containing 60% kcals from fat with 5.24 kcals/g (DIO series D12492, Research Diets Inc.). Groupings included control- and high-fat-fed (HF-fed) males and females that were monitored for food intake, bodyweight, and behavior during NIH. A HFD replacement paradigm started 40 days into feeding, during which HF-fed groups had their HFD replaced with control diet and control groups were pair fed their normal diet. Half of the mice proceeded to EPM testing and FSCV one day after HFD replacement, and the other half proceeded one week after HFD replacement. NIH testing was used to assess baseline food-related anxiety behaviors induced by consuming a HFD, whereas the EPM test was used to assess whether stress induced by HFD replacement promoted general anxiety-like behaviors in addition to baseline food-specific anxiety. Further, NIH was conducted during HFD intake because it is a more prolonged test requiring training that could confound effects of HFD replacement, whereas the EPM is much faster and allowed for assessment of anxiety after HFD replacement on the same day that neurochemistry was evaluated. Final sample sizes included *n* = 9 per diet X sex X HFD replacement group (*N* = 72) (Figure 1a).

### 2.2. Novelty-Induced Hypophagia (NIH)

To characterize the effect of a HFD on the anxiety-promoting effects of a novel environment, we conducted the NIH test adapted from Dulawa (2009) [22] and Browne et al. (2018) [23] during the sixth week of feeding. NIH training began between weeks four and five with exposure to a metal food cup containing 7 g of sucrose cubes (30 kcals) for 30 min each day over three days. All training and test sessions were completed between 0830 and 1130 at the beginning of dark cycle with sessions recorded via HomeCageScan (v.3) software. Training occurred under low red-light conditions in similar cages to the home cage with normal bedding. Mice were transferred to the behavioral room in the home cage covered by an opaque filter lid to prevent exposure to bright light during training, and each mouse had its own separate cage that was used for all training sessions without changing bedding or food cups to promote familiarity with the training environment. Approximately four days after the final training session, the NIH test was performed, during which mice were placed into a new cage without bedding sitting atop white paper in a brightly lit room, which created novelty related to use of a clean cage and clean food cup with high light contrast during the normal dark cycle. Mice were given sucrose (7 g) in a clean cup and monitored for 30 min. Sucrose consumption was determined by weighing initial and remaining sucrose. A Latin Square design was used to counterbalance the order of training and testing between groups. Video recordings were analyzed with TopScan (v.2) software to assess latency to approach the sucrose cup. Mice returned to normal feeding conditions for four days before HFD replacement.

### 2.3. HFD Replacement and the Elevated Plus-Maze (EPM)

To examine whether removal of a preferred HFD caused anxiety-like behaviors, HF-fed mice were switched to the low-fat control diet. This induced voluntary caloric restriction, so controls were pair fed their normal control diet to match intake and weight loss in HF-fed mice. Pair-feeding ensured group differences in EPM and FSCV were due to palatable food removal rather than voluntary caloric restriction and weight loss in HF-fed groups. Because mice were group housed and control food pellets weighed a similar amount as control groups were fed each day, pellets were crushed and delivered to mice in pieces divisible by three to ensure an equal number of crushed pellets per mouse. Control mice were fed once per day between 0900 and 1000, during which observations were made to ensure each mouse was eating. This timing matched the onset of NIH, EPM, and FSCV experiments. Half of the mice (*n* = 36) were maintained on control diet for one week before proceeding to the EPM and ex vivo slice FSCV, and a second group of mice (*n* = 36) underwent the same paradigm but proceeded to EPM and FSCV experiments after one day to capture acute effects of HFD replacement. Because HF-fed males and females in the one-day HFD replacement condition ate, on average, less than 0.2 kcal per mouse, controls were fasted for one day. Control mice in the one-week HFD replacement condition were pair fed based on percent bodyweight loss and did not significantly differ in weight loss (*p* > 0.15), whereas controls lost a greater percentage of weight than HF-fed counterparts in the one-day condition (*p* < 0.0001). More information about kcal intake and bodyweight loss during HFD replacement and pair-feeding is available in Supplementary Figure S1.

EPM experiments were adapted from Sharma and Fulton (2013) [13] and Pawlak et al. (2012) [24] to assess spontaneous avoidance behavior indicative of anxiety. We used an opaque plexiglass maze raised 60 cm from the ground with a 10 × 10 cm square base with one arm coming from each side. Two of the arms opposite each other were open (10 × 32.5 cm) while the other two arms were closed (10 × 32.5 × 15 cm) with 15 cm high walls. Mice were placed on the EPM at 0900 and allowed to explore for five minutes under video recording using HomeCageScan (v.3) software. TopScan (v.2) was used to measure the percent duration in the open, closed, and square intersection of both arms.

### 2.4. Fast-Scan Cyclic Voltammetry (FSCV)

Immediately following the EPM, mice proceeded to ex vivo FSCV. Mice were anesthetized via 5% isoflurane and then decapitated for brain removal. Brains were placed in oxygenated (95% O_2_/5% CO_2_) artificial cerebrospinal fluid (aCSF) composed of (NaCl 126 mM, NaHCO_3_ 25 mM, D-glucose 11 mM, KCl 2.5 mM, CaCl_2_ 2.4 mM, MgCl_2_ 1.2 mM, NaH_2_PO_4_ 1.2 mM, L-ascorbic acid 0.4 mM, pH adjusted to 7.4) then sliced into 300 μm coronal slices between +0.9 and +1.5 mm from Bregma with a compresstome (Precisionary Instruments; Greenville, NC). Experiments proceeded after 60 min of equilibration in oxygenated aCSF (100 mL/h) using a glass capillary-pulled carbon fiber recording electrode placed 75 μm within the slice next to a bipolar stimulating electrode. Dopamine release was evoked and recorded for 15 s by a single monophasic electrical pulse (350 μA, 4 ms) occurring every 180 s until baseline dopamine recordings were stable (<10% change) between three or more recordings. Dopamine was measured by the recorder scanning with a triangular waveform between −0.4 V and 1.2 V against Ag/AgCl reference at a rate of 400 V/s every 100 ms. Dopamine current (nA) was converted to concentration (μM) using electrode calibrations with 3 μM dopamine solution after each experimental day. Recording and analysis were performed using DEMON Voltammetry and Analysis Software [25]. Analysis included modeling recordings with Michaelis−Menten kinetics to determine maximal [dopamine] and dopamine reuptake (V_max_) while holding Km (dopamine affinity for DAT) constant at 160 nM.

Pharmacological manipulations occurred in each slice after baseline recordings, which were taken in the NAc core ventrally to dorsal-medially of anterior commissure based on KOR expression patterns [15,19]. After stabilization of dopamine signals, a dose response curve was conducted in a cumulative manner by adding 0.01, 0.10, and 1.0 µM of KOR agonist (-)-U-50488 hydrochloride (U50) (Tocris; Bristol, UK; Cat. No. 0496) to the aCSF. Pharmacological investigation was also conducted in separate slices using 1.0 µM KOR antagonist nor-Binaltorphimine dihydrochloride (norBNI) (Tocris; Bristol, UK; Cat. No. 0347) followed by U50 (0.10 µM). These doses were based on previous studies [26,27] that used U50 and norBNI.

### 2.5. Statistical Analysis

Statistical analysis was conducted via GraphPad Prism (v. 9.0.0). Two-way analysis of variance (ANOVA) was used to identify diet or sex effects in daily food intake and NIH outcomes. Within each sex, two-way repeated-measures ANOVAs identified effects of diet over time on bodyweight. Repeated-measures two-way ANOVAs were also used to assess differences in food intake before and after HFD replacement in HF-fed mice within the one-day or one-week HFD replacement time conditions. Baseline dopamine release and V_max_ were assessed within each HFD replacement condition by two-way ANOVA. Two-way ANOVAs were further used to identify anxiety-like behavior during the EPM and for effects of norBNI versus U50 on dopamine release within either sex and HFD replacement time condition, and repeated-measures was used specifically to identify effects of dose during the U50 curve. All group differences were assessed using Šidák’s or Tukey’s post hoc tests. Results are expressed as the mean ± standard error of the mean.

## 3. Results

### 3.1. Chronic HFD Feeding Promoted Caloric Intake and Weight Gain

A two-way ANOVA revealed effects of diet (F(1,20) = 81.27; *p* < 0.0001) and sex (F (1,20) = 11.13; *p* < 0.01) on energy intake, and mice fed the HFD consumed more energy each day than controls (males: 14.2 ± 0.3 vs. 10.6 ± 0.4 kcals/day; *p* < 0.0001) (females: 13.1 ± 0.6 vs. 8.9 ± 0.3 kcals/day; *p* < 0.0001) (Figure 1b). This corresponded with significant effects of diet (F(1,34) = 49.46; *p* < 0.0001), time (F(1,34) = 457.2; *p* < 0.0001), and subject (i.e., each mouse) (F(34,34) = 3.018; *p* = 0.0009) with a diet x time interaction (F(1,34) = 168.9; *p* < 0.0001) on bodyweight in males with higher bodyweights in HF-fed mice compared to controls by week five (36.2 ± 0.8 vs. 26.6 ± 0.5 g; *p* < 0.0001) (Figure 1c). Similarly, in females, there were effects of diet (F(1,34) = 35.07; *p* < 0.0001), time (F(1,34) = 152.5; *p* < 0.0001), and subject (i.e., each mouse) (F(34,34) = 2.252; *p* = 0.0102) with a diet x time interaction (F(1,34) = 62.09; *p* < 0.0001) and greater bodyweight in HF-fed versus control females at week five (26.8 ± 1.0 vs. 19.5 ± 0.3 g; *p* < 0.0001) (Figure 1d).

### 3.2. HFD Consumption Reduced Sucrose Intake during Novelty-Induced Hypophagia

**Figure 2:**
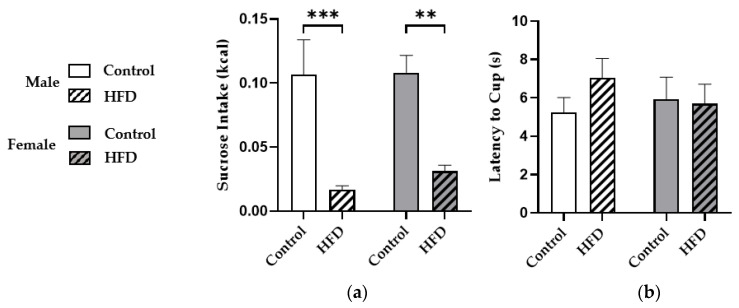
Sucrose Consumption and Latency to Approach Sucrose during the Novelty-Induced Hypophagia (NIH) Test. Sucrose intake in kcals (**a**) and latency to approach the sucrose cup in seconds (**b**) during the NIH test. * denotes a significant difference between diet groups. **, *p* < 0.01; ***, *p* < 0.001.

Next, we performed the NIH test to assess anxiety during palatable food access. A two-way ANOVA with males and females showed a main effect of diet (F(1,66) = 30.25; *p* < 0.0001) but not sex (*p* = 0.5936), and mice consuming HFD consumed significantly less sucrose during the NIH test than controls in males (0.017 ± 0.003 vs. 0.107 ± 0.027 kcals; *p* = 0.0002) and females (0.031 ± 0.005 vs. 0.108 ± 0.013 kcals; *p* = 0.0011) (Figure 2a). However, no significant effects of diet (*p* = 0.4366) or sex (*p* = 0.7471) on latency to approach sucrose were observed (Figure 2b).

### 3.3. Replacing HFD with Control Diet Induced Voluntary Caloric Restriction but General Anxiety-Like Behaviors Were Similar to Controls

**Figure 3:**
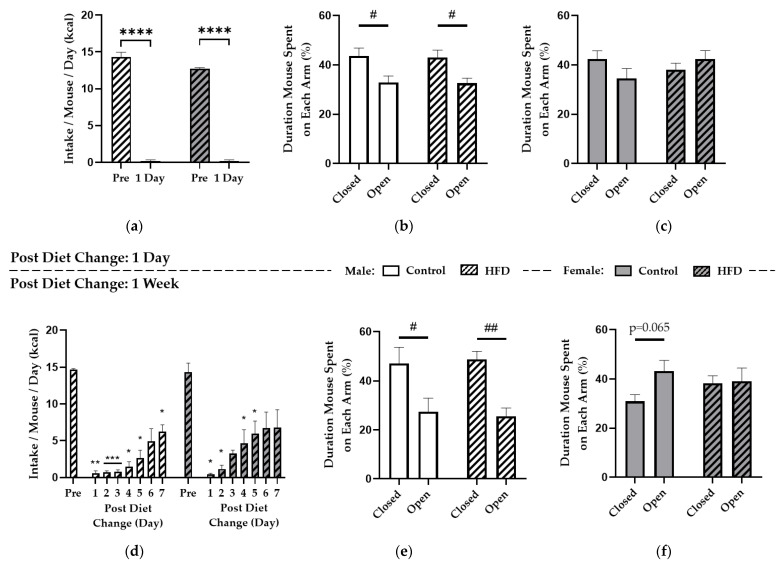
Effect of HFD Replacement on Control Diet Consumption in HF-Fed Mice and Anxiety-Like Behaviors Determined by the Elevated Plus-Maze (EPM). One day after HFD replacement, HF-fed males and females almost completely restricted kcal intake (**a**). EPM results show the total percent duration spent on the closed versus open arms for males (**b**) and females (**c**) one day after HFD replacement or pair-feeding in controls. Intake for HF-fed males and females is also shown over one week of HFD replacement (**d**) with EPM results displayed as percent duration on closed versus open arms for males (**e**) and females (**f**). * denotes a significant difference between kcal intake in HF-fed mice before (Pre) and each day after HFD replacement; # denotes significant differences between time spent on the closed versus open arms of the EPM. *, *p* < 0.05; **, *p* < 0.01; ***, *p* < 0.001; ****, *p* < 0.0001; #, *p* < 0.05; ##, *p* < 0.01.

After NIH, all mice entered the HFD replacement paradigm. Two-way repeated-measures ANOVA revealed significant effects of time (F(1,4) = 913.4; *p* < 0.0001) and sex (F(1,4) = 8.180; *p* = 0.0459) on food intake one day after HFD replacement, and post hoc tests showed prior HFD kcal consumption was greater than control diet intake in HF-fed males (14.3 ± 0.7 vs. 0.2 ± 0.2 kcals/day; *p* < 0.0001) and HF-fed females (12.7 ± 0.2 vs. 0.2 ± 0.2 kcals/day; *p* < 0.0001) (Figure 3a). Further, the sex effect on intake was driven by greater average kcal intake of HFD the week prior to HFD replacement by males versus females (*p* = 0.0326), but there was no difference in response to intake of control diet. One day into the HFD replacement paradigm or pair-feeding in controls, EPM testing revealed no effect of diet (*p* = 0.8635), but there was a main effect of EPM arm in males (F(1,44) = 15.45; *p* < 0.001) with more time spent on the closed versus open arms for control (43.7 ± 3.1 vs. 32.9 ± 2.6%; *p* = 0.0145) and HF-fed males (43.1 ± 2.9 vs. 32.6 ± 2.1%; *p* = 0.0174) (Figure 3b). Conversely, females did not exhibit an effect of diet (*p* = 0.6027) or EPM arm (*p* = 0.6147) (Figure 3c). Therefore, replacing a HFD with control diet for one day induced near-total caloric restriction similarly in males and females but promoted outward anxiety-like behaviors specifically in males, whereas anxiety was unchanged between diet groups with no greater effect induced by HFD replacement compared to control pair-feeding. 

Similar to one day of HFD replacement, two-way repeated-measures ANOVA revealed prolonged voluntary food restriction over a full week, with significant effects of time (F(1.833,7.334) = 40.75; *p* = 0.0001) and subject (i.e., average kcal intake per mouse for each cage) (F(4,28) = 5.473; *p* = 0.0022) but not sex (*p* = 0.2945) (Figure 3d). Intake of control diet following HFD was significantly reduced in males on days one, two, three, four, five, and seven and similarly in females on days one, two, four, and five. During EPM testing after one week of HFD replacement or pair-feeding in controls, there was no effect of diet (*p* = 0.9810), but there was a significant effect of EPM arm in males (F(1,20) = 19.64; *p* < 0.001). Further, post hoc testing revealed more time spent in the closed versus open arms for controls (47.2 ± 6.5 vs. 27.4 ± 5.5%; *p* = 0.0183) and HF-fed males (48.8 ± 3.2 vs. 25.6 ± 3.3%; *p* < 0.01) (Figure 3e). However, no effect of diet (*p* = 0.7007) or EPM arm (*p* = 0.1152) were reported for females (Figure 3f). Therefore, males exhibited anxiety-like behaviors after either one day or one week of dietary change, whereas females did not exhibit any anxiety-like behavior. Overall, because EPM response did not differ between diet groups, results indicate that HFD intake altered preference for an alternate palatable treat, via NIH, whereas HFD replacement induced food-related anxiety indicated by reduced food intake but did not affect general anxiety during the EPM test.

### 3.4. After HFD Replacement, Dopamine Release and Reuptake in the NAc Core Were Similar to Controls

**Figure 4:**
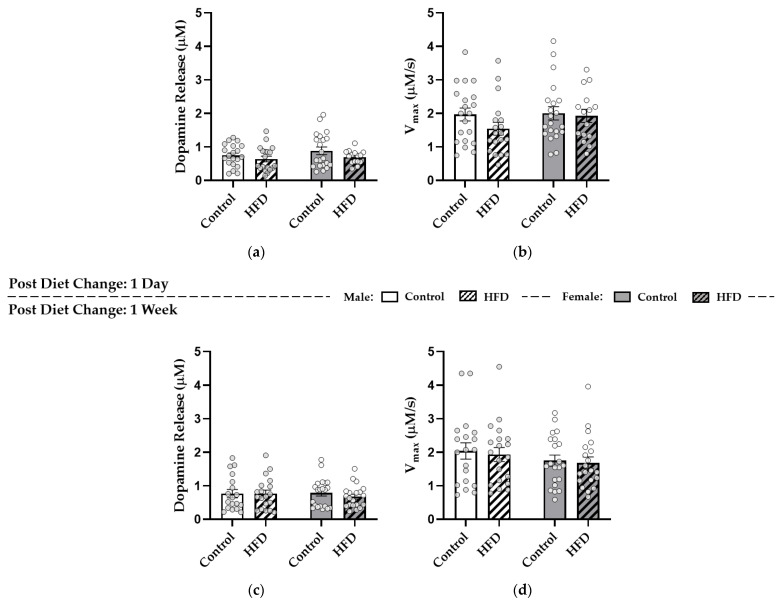
Effect of HFD Replacement on Baseline Dopamine Release and Reuptake within the NAc Core. Dopamine release evoked by a single electrical pulse (μM) and maximal rate of reuptake (V_max_) (μM/s) after one day (**a**,**b**) or one week (**c**,**d**) of HFD replacement. No significant effects were reported.

Immediately after the EPM, mice entered ex vivo FSCV experiments to assess dopamine neurotransmission. Two-way ANOVAs were conducted to assess effects of diet and sex within either the one-day or one-week conditions for both “tonic” dopamine release evoked by stimulation with a single electrical pulse and V_max_. After one day of HFD replacement or pair-feeding in controls, there were no significant effects of diet (*p* = 0.1031) or sex (*p* = 0.2698) on dopamine release (Figure 4a) or of diet (*p* = 0.2106) or sex (*p* = 0.2906) on V_max_ (Figure 4b). After one week of HFD replacement or pair-feeding, there were also no effects of diet (*p* = 0.5739) or sex (*p* = 0.7471) on dopamine release (Figure 4c) or of diet (*p* = 0.6673) or sex (*p* = 0.1815) on V_max_ (Figure 4d).

### 3.5. Enhanced κ-Opioid Receptor-Mediated Control over Dopamine Release in the NAc Core Was Observed One Day after HFD Replacement with Control Diet in Females

**Figure 5:**
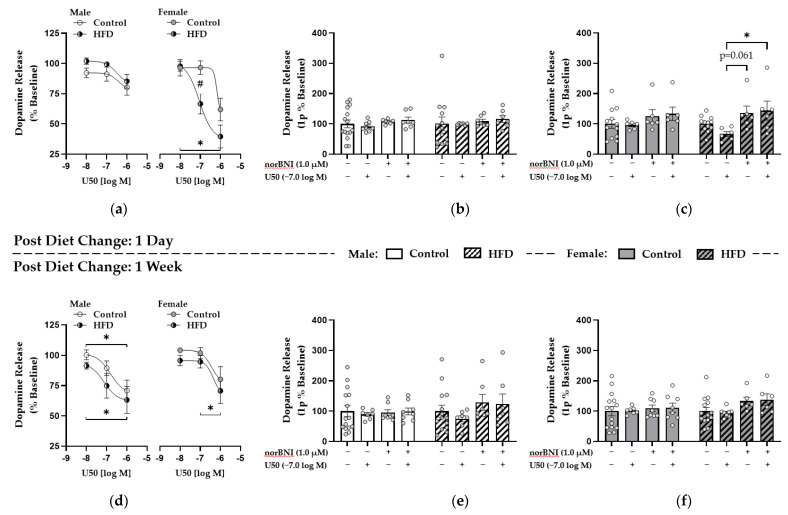
κ-Opioid Receptor (KOR)-Mediated Control Over Dopamine Release within the NAc Core During HFD Replacement. Effects of KOR agonism with (-)-U-50488 hydrochloride (U50) in males and females after one day of HFD replacement or pair-feeding in control groups (**a**) are displayed using 0.01 μM (−8.0 [log M]), 0.10 μM (−7.0 [log M]), and 1.0 μM (−6.0 [log M]) U50. Interactions between KOR agonism with 0.10 μM U50 and KOR antagonism with 1.0 μM nor-Binaltorphimine dihydrochloride (norBNI) are shown for males (**b**) and females (**c**). Similarly, for the one-week HFD replacement and pair-feeding condition, the U50 drug curve is shown for males and females (**d**) with interactions between U50 and norBNI shown for males (**e**) and females (**f**). * denotes significant differences between U50 doses or between effects of U50 and norBNI; # denotes a significant diet effect within an individual dose of U50. *, *p* < 0.05; #, *p* < 0.05.

After establishing stable dopamine recordings, a KOR agonist (U50) and antagonist (norBNI) were applied to probe KOR functionality. Dopamine release is reported as a percent of the pre-drug baseline recording for the U50 curve and 1.0 μM norBNI. One day into HFD replacement or pair-feeding in controls, there was a main effect of U50 on dopamine release in males (F(1.577,18.92) = 11.21; *p* = 0.0012) and a significant U50 dose effect per subject (i.e., each brain slice) (F(12,24) = 5.033; *p* = 0.0004), but no effect of diet (*p* = 0.2283) was found (Figure 5a). In females, we observed main effects of U50 dose (F(1.571,15.71) = 22.12; *p* < 0.0001) and diet group (F(1,10) = 6.084; *p* = 0.0333), with post hoc analysis indicating a dose-dependent decrease in dopamine release in HF-fed females (*p* < 0.05) and significantly lower dopamine release in the HF-fed females compared to controls at −7.0 [log M] U50 (66.6 ± 8.4 vs. 96.4 ± 5.5%; *p* = 0.0478) (Figure 5a). To determine whether changes in dopamine release were due to activation of KORs, we applied the KOR antagonist norBNI (1.0 μM) prior to 0.1 μM U50 and compared this to the 0.1 μM dose from the U50 drug curve. After one day of HFD replacement or pair-feeding in controls, a two-way ANOVA in males revealed no significant drug (*p* = 0.6781) or diet (*p* = 0.7785) effects on dopamine release (Figure 5b). However, in females, there was a significant effect of drug (F(3,52) = 4.138; *p* = 0.0105) but not diet (*p* = 0.8605), and Tukey’s multiple comparisons test revealed reduced dopamine release specifically in HF-fed females at −7.0 [log M] U50 compared to 1.0 μM norBNI and when U50 was added after norBNI (66.6 ± 8.4 vs. 135.6 ± 23.1 vs. 143.8 ± 31.3%; U50, norBNI, norBNI + U50, respectively; *p* = 0.0614, *p* = 0.0291) (Figure 5c). Therefore, receptor specificity was shown with the ability of KOR-specific antagonism to block KOR-mediated reductions in dopamine release.

### 3.6. κ-. Opioid Receptor Function Was Upregulated in Males after One Week of HFD Replacement while These Effects Were Attenuated in Females

After one week, males had a significant effect of dose (F(1.446,17.36) = 19.85; *p* = 0.0001) and subject (i.e., each brain slice) (F(12,24) = 6.643; *p* < 0.0001) but not of diet (*p* = 0.2999) for the U50 curve, with significantly reduced dopamine release between −8.0 and −6.0 [log M] U50 for control (100.4 ± 4.0 vs. 71.0 ± 8.6%; *p* = 0.0161) and HF-fed males (91.5 ± 2.8 vs. 63.2 ± 11.1%; *p* = 0.0419) (Figure 5d). Conversely, effects were weaker in females after one week, with an effect of dose (F(1.266,16.46) = 11.93; *p* = 0.0019) and subject (i.e., each brain slice) (F(13,26) = 2.654; *p* = 0.0165) but not of diet (*p* = 0.2848), and post hoc tests revealed reduced release between only −7.0 and −6.0 [log M] U50 for HF-fed females (94.7 ± 5.1 vs. 70.7 ± 10.4%; *p* = 0.0361) (Figure 5d). Further, two-way ANOVA during the norBNI and U50 comparison revealed no significant effects of drug (*p* = 0.4919) or diet (*p* = 0.4388) in males (Figure 5e) or of drug (*p* = 0.1639) or diet (*p* = 0.3048) in females (Figure 5f) after one week. Overall, KOR sensitivity was upregulated in males after one week of HFD replacement, indicated by the U50-induced reduction in dopamine release, whereas females showed enhanced KOR effects on dopamine terminals after one day with U50 reducing dopamine release and norBNI blocking this reduction specifically in HF-fed females.

## 4. Discussion

This study sought to characterize food and anxiety-like behaviors and KOR-mediated control over dopamine release in the NAc core in mice accustomed to consuming a HFD after replacing it with a less preferred, low-fat control diet. We show that (1) consuming a HFD significantly reduced sucrose intake, suggesting palatable inflexibility and reduced food intake under anxiogenic feeding conditions; (2) replacing a HFD with a low-fat option induced voluntary caloric restriction that persisted for several days; (3) removal of a preferred palatable food in HF-fed mice did not induce anxiety-like behaviors during the EPM compared to controls; and (4) sensitivity of KORs to an exogenous ligand that reduced dopamine release in the NAc core was upregulated in females after one day but in males after one week of HFD replacement. This is the first study to support that KORs are directly involved in reducing tonic dopamine neurotransmission in the NAc core specifically after abruptly switching an animal accustomed to high-fat food to a less palatable option lower in energy density. Further, we reported a greater acute response to KOR agonism in females that was blocked by receptor antagonism with norBNI, whereas there was a more protracted response in males. Overall, HFD intake promoted dietary inflexibility toward another palatable option, and changes in KOR function were associated with voluntary reduction in food intake during HFD replacement.

While no other investigation has assessed effects of KOR stimulation on NAc dopamine after abrupt dietary changes, other studies have considered effects of palatable food removal. As reported herein, HFD-induced sucrose anhedonia has been observed in males [4] and females [28], and replacement of a HFD or Western diet (WD) (a HFD with sugar) with chow [3,4] reduced food intake. Further, removal of the preferred diet increased motivation to obtain sucrose and fat and heightened activation of the hypothalamic–pituitary–adrenal (HPA) axis upon stress exposure [3,4]. EPM testing showed increased anxiety-like behavior only in HF-fed mice when groups fed a control low-fat diet or HFD had their diets replaced with ad libitum chow for one day [4]. In contrast, we reported that both control and HF-fed males exhibited anxiety-like behavior during the EPM at one day and one week, suggesting that general anxiety during the HFD replacement paradigm stemmed from a negative energy state and not specifically from replacing HFD with a less preferred option. Our hypotheses that HFD intake would induce sucrose anhedonia and HFD replacement would reduce consumption of a new, less palatable diet were supported but, although HF-fed males took slightly longer to approach sucrose during NIH, we did not report significant diet effects on latency. Conversely, latency to consume chow was increased after HFD feeding during NIH [29], suggesting that HFD intake herein did not alter anxiety due to a novel environment but similar latency to approach sucrose between diet groups could be due to greater salience for sucrose compared to chow. It is possible that KORs could account for these effects. Indeed, ventricular U50 administration promoted HFD intake in sated rats, whereas norBNI inhibited HFD intake in a fasted state with no effect on low-fat diet intake [30]. Similarly, 16 h fasted mice with systemic norBNI injection reduced chow intake while NAc-specific injection of a non-selective opioid antagonist reduced food intake [31]. Further, systemic KOR agonism reduced phasic dopamine release in the NAc core in vivo that paralleled reductions in motivation to obtain sucrose [32]. These studies support an important function of KORs in the NAc core in controlling dopamine release to alter rewarding effects of food but to increase motivation to obtain and consume palatable foods. Results herein support that replacing HFD with a low energy-dense option promoted KOR sensitivity that reduced dopamine tone associated with dietary inflexibility without affecting general anxiety-like behavior.

Food restriction and weight loss also affect the DAT and KORs. While reduced dopamine reuptake V_max_ is a hallmark finding due to chronic HFD intake [9,11], this effect was not significant after HFD replacement. It is possible that a reduction in intake and bodyweight plus food-related stress negate this diet-induced effect. For example, 12 h of food deprivation restored the reduction in dopamine reuptake V_max_ in the NAc exhibited by ad libitum HF-fed versus control mice [33]. Conversely, food restriction and obesogenic diet intake reduced tonic dopamine release and V_max_ compared to controls in the NAc core, which was restored by insulin in food restricted rats [34], and food restriction promoted locomotor activity induced by psychostimulants [35]. Overall, evidence from these studies suggest that food restriction promotes sensitivity of DAT shuttling and function, and these effects may be further explained by alternate receptor systems. For example, food restriction promoted locomotor activity in response to ventricular injection of D1R and D2R agonists compared to ad libitum counterparts, whereas D2R agonism reduced activity compared to vehicle during ad libitum feeding [36]. This difference in behavioral response specifically to D2R agonism between ad libitum feeding and food restriction suggests that negative energy states alter the ratio of pre- and postsynaptic D2Rs. Finally, a 16 h fast reduced prodynorphin expression in the NAc and ventral tegmental area [31], while a 72 h fast reduced striatal dynorphin protein concentrations in lean but not obese rats [37], effects which could explain why males on the control diet showed increased sensitivity to U50 after one week of pair-feeding. Indeed, promotion of endogenous KOR ligands by drug abuse [38] and stimulation by exogenous KOR agonists [39] both reduced KOR expression in systemic tissues, whereas KOR antagonism increases KOR expression [40], suggesting endogenous KOR ligand concentrations direct KOR expression and sensitivity. However, in human alcoholics, cortical expression of prodynorphin positively correlated with dynorphin and KOR expression [41]. Further, NAc dynorphin and KOR expression were not affected by ad libitum or intermittent HFD access [42], WD intake, nor WD replacement with chow [43]. Tied to the fact that we reported greater effects of KOR stimulation in HF-fed mice, these results together support that HFD replacement promoted KOR-mediated control over dopamine release rather than altering expression patterns. KORs colocalize with DAT-expressing dopamine axon terminals [19] and reduce synaptic dopamine by promoting DAT shuttling via ERK1/2 signaling [17] similarly to D2R autoreceptors and insulin [18], and food restriction increased NAc insulin receptor and D2R expression [44]. These reports suggest that energy restriction promotes D2R autoreceptor and insulin receptor sensitivity or expression, reduces expression of KOR ligands, but promotes function or sensitivity of KORs in the NAc core. These effects of food restriction together could promote KOR function and DAT shuttling and explain why we did not report reduction in V_max_ in HF-fed mice during HFD replacement.

Prior evidence supports that HFD replacement reduces food consumption but increases anxiety, and our data support that these effects could be due to KORs. However, few studies have included females. Whereas NAc prodynorphin and KOR expression are similar between sexes [45], sex effects have been reported in response to diet. For example, males exhibited heightened diet-induced striatal insulin resistance, a reduction in dopamine release and reuptake, and D2R sensitivity [11,34,46]. Differences also exist in response to removal of preferred foods. HFD intake from birth through 12 weeks reduced NAc D1R and D2R expression, whereas four-week replacement with control diet further reduced expression in males but restored expression in females [28]. However, when females were fed HFD from age six to 18 weeks followed by four-week HFD replacement [47], HFD-induced reduction in D2R expression did not recover. It is possible that KOR-mediated effects may be delayed in males during HFD replacement due to longer recovery of insulin sensitivity and dopamine receptor systems and a possible ceiling effect of D2R autoreceptor-mediated reduction in tonic dopamine. Conversely, high-choice cafeteria diet consumption for eight weeks reduced NAc tyrosine hydroxylase expression in males, but increased tyrosine hydroxylase in females, effects that were not restored by three days of diet removal [48]. This suggests that heightened effects of KORs in females herein could stem from dopamine synthesis during acute HFD replacement. Alternately, D1R expression was increased by cafeteria diet and reduced by diet removal in males but cafeteria diet reduced NAc D1R in females that was not restored [48]. KORs are expressed on D1R-expressing GABAergic medium spiny neurons [49], and D1R activation induces dynorphin release [14]. This suggests that HFD-induced downregulation of D1Rs in females could acutely sensitize KORs. Females may also be more sensitive to acute HFD replacement because of effects on the DAT as evidenced by increased efficacy of U50 in females to block locomotor effects of psychostimulants [50]. However, females exhibited lower sensitivity of KOR agonism to induce anhedonia, analgesia, and NAc ERK1/2 phosphorylation and reduce NAc dopamine release [45,51] and to norBNI-induced JNK activation [52] linked to estrogen-dependent activation of GRK2. Because KOR-mediated intracellular signaling is less sensitive in females, further investigation is required to explain why we reported greater acute effects of HFD replacement in females. A heightened stress response to HFD replacement might also account for this effect, as prepubertal HFD intake promoted HPA axis response more strongly in females [53]. Conversely, repeated stress in male rats increased NAc dynorphin and KOR expression after nine but not two days of recovery [54], supporting increased KOR function in males after one week of HFD replacement. Overall, few studies have assessed diet and sex interactions within NAc KOR-mediated signaling, so further investigation is required to determine mechanisms underlying sex effects reported herein.

This study had several strengths, including use of both sexes, assessment of anxiety during HFD intake and immediately before FSCV during HFD replacement, and investigation of KOR function after stressful dietary changes not previously reported. Herein, KOR sensitivity was acutely upregulated specifically in HF-fed females with dose-dependent effects of U50 to reduce dopamine release and stimulation of release by norBNI after one day of HFD replacement. Conversely, males exhibited dose-dependent reduction in dopamine release by U50 only after one week, with greater reduction in HF-fed mice. One limitation involved a lack of control and HFD groups that did not undergo HFD replacement by maintaining ad libitum food access. However, our lab previously showed that, whereas ad libitum HFD intake inhibited phasic dopamine release and reuptake [9], food deprivation restored these effects [33]. The HFD replacement and pair-feeding paradigm herein is different than total food deprivation but similarly produced a negative energy state, supporting the present results that there were no baseline differences in NAc dopamine neurochemistry between groups at one day or one week of HFD replacement. In comparison, U50 reduced dopamine release in control and HFD groups, but there was a stronger effect in HF-fed females after one day of HFD replacement. Therefore, because there were no differences in baseline dopamine release induced by a negative energy state, but KOR agonism had an effect to reduce dopamine release more strongly in HF-fed groups at certain timepoints, this evidence supports that the present comparisons were fair.

## 5. Conclusions

In conclusion, HFD intake induced sucrose anhedonia during NIH, whereas replacing the preferred HFD with a low energy-dense option promoted food-related, but not general, anxiety-like behavior exhibited by voluntarily reduced intake and further promoted KOR sensitivity to reduce NAc dopamine release. Greater sensitivity or function of KORs would reduce dopamine tone in the NAc, and this reduction in basal dopamine could produce a greater magnitude of difference in synaptic dopamine concentration due to phasic burst firing of dopamine neurons upon exposure to palatable foods. This greater relative increase in synaptic dopamine from phasic release after reduction in dopamine tone by KORs could promote hedonic valuation of energy-dense foods during a negative energy state, possibly leading to overeating to restore dopamine concentrations. This upregulation of KOR sensitivity could, theoretically, hinder weight loss due to greater salience of palatable foods. While the preliminary findings presented herein are limited to rodent experiments, upregulation of KOR function in response to diet-related stressors that reduce dopamine tone might translate to obese individuals who limit energy intake for weight loss. Further study is required to determine mechanisms responsible for promoting KOR sensitivity in the NAc as well as the safety and efficacy of using KOR-modulating drugs to promote the success of medically supervised weight loss interventions.

## Data Availability

Data are available from S.C.F. upon request.

## Supplementary Materials

The following are available online at https://www.mdpi.com/article/10.3390/nu13072341/s1, Figure S1: Comparison of Intake and Bodyweight Loss during HFD Replacement vs. Pair-Feeding of Controls.
