# Supplementary material for: Replacing a Palatable High-Fat Diet with a Low-Fat Alternative Heightens κ-Opioid Receptor Control over Nucleus Accumbens Dopamine
Source: Nutrients. 2021 Jul 9;13(7):2341. doi: 10.3390/nu13072341 (PMC8308677; doi:10.3390/nu13072341)
Supplement: Supplementary file 1 [file nutrients-13-02341-s001.zip › nutrients-1221596-supplementary.pdf]

Supplementary Materials

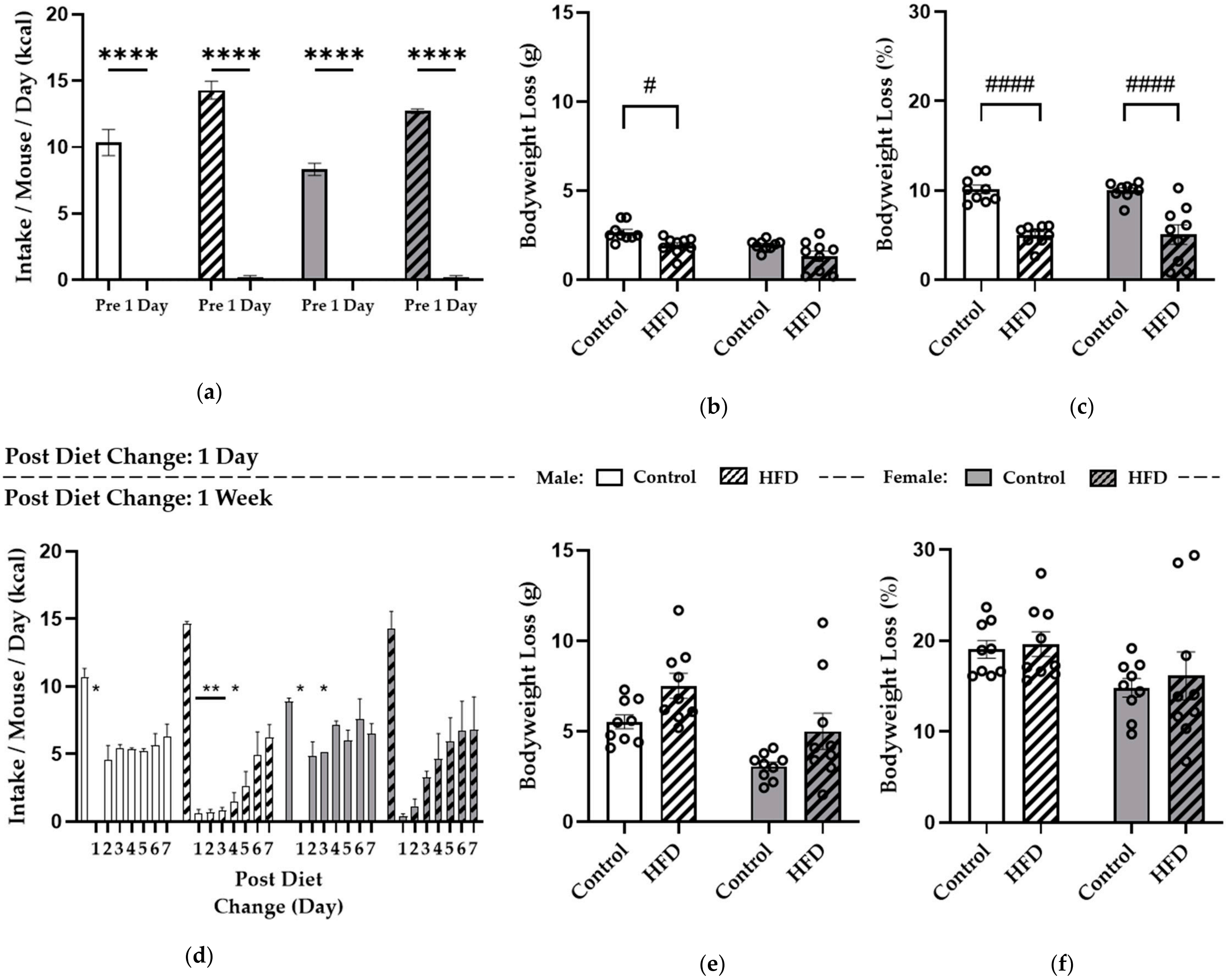

Figure S1. Comparison of Intake and Bodyweight Loss During HFD Replacement vs. Pair-Feeding of Controls – Male and female data is displayed one day into HFD replacement or pair-feeding in controls for intake (kcal) (a) and bodyweight loss in grams (b) and percent bodyweight (c). Data is displayed in a similar way after one week of HFD replacement or pair-feeding in controls for intake (kcal) (d) and bodyweight loss in grams (e) and percent bodyweight (f). Individual values are shown for all mice for bodyweight loss. Intake data was compared between all diet X sex groups over time with significant differences only displayed within group, whereas bodyweight loss was compared between diet groups. (* denotes a significant difference between kcal intake before (Pre) and each day after HFD replacement, # denotes differences between diet groups in bodyweight loss) (*, $p < 0.05$; **, $p < 0.01$; ****, $p < 0.0001$; #, $p < 0.05$; ####, $p < 0.0001$).
